# Marital Surname Change and Marital Duration Among Divorcées in a Canadian County

**DOI:** 10.3389/fpsyg.2021.647942

**Published:** 2021-06-16

**Authors:** Melanie MacEacheron

**Affiliations:** Department of Psychology, University of Western Ontario, London, ON, Canada

**Keywords:** divorce, marital surname change, marriage duration, number of children of the marriage, Canada

## Abstract

Women’s marital surname change has been discussed as comprising one possible signal of intention to remain married, and may be perceived as such, and valued, by husbands. Here, the practice was investigated as a potential predictor of marital duration among couples who went on to divorce. An archival analysis was based on a search of all available, opposite-sex divorces filed over an 8-month period in a Canadian county. Among couples (*n* = 107) divorcing, marriages the women in which underwent marital surname change lasted 60% longer, controlling for wife’s age at the time of marriage. When the woman’s marital surname change/retention was used as a regression predictor of number of children of the marriage alongside marriage duration in years, only the latter was predictive. No husband took his wife’s surname. Giving the maternal surname (along with the paternal surname) to children occurred at a negligible frequency. Potential reasons for these findings including costly signaling and, ultimately, paternity uncertainty, as well as possible implications for public policy, are discussed.

## Introduction

In countries such as Canada and the US, women, and only women, customarily change surname at marriage to that of their male spouses despite surname retention being the default and easier option. In fact, only 6% (Gooding and Kreider, 2010) or 4.6% (Johnson and Scheuble, 1995) of US women may choose non-traditional marital surnaming (analogous data unavailable for Canada). Reasons for the custom may include such change comprising one, possible, costly signal of marital commitment, which is on average desired by men (MacEacheron, 2016a, 2020: regarding costly signaling generally, see Nesse, 2001). Yet some married women continue to use their premarital surnames. There is evidence that men care whether their wives take their surname (for instance, possessing this as an expectation: Lockwood et al., 2011), as well as, perhaps, whether their children are surnamed for them (e.g., Cherlin, 1978: see also Robnett and Leaper, 2013). Marital surname choice is reported to sometimes be a fraught decision (e.g., Boxer and Gritsenko, 2005).

Marital surname choice has been reported as seen by women as potentially negatively impacting women’s earnings (Goldin and Shim, 2004). Yet, it has been viewed as particularly appropriate for couples planning children (Scheuble and Johnson, 1998) – an expensive enterprise. There is also evidence of largely negative perceptions of women based on their retention or hyphenation of premarital surnames. Such perceptions include that they possess attributes and behavior that may lead to divorce or poorer mothering (e.g., Stafford and Kline, 1996; Murray, 1997; Suter, 2004). These facts beg two questions, inspiring the current research: (1) Is this decision associated with marital duration? (2) Is this decision associated with the number of children of the marriage?

Murray (1997) observed that American men expressed the view that women retaining/hyphenating surname at marriage are less attractive and make worse mothers. As for women who hyphenate their surnames with those of their husbands, one study found that American undergraduates perceived them as relatively “career oriented” with men scoring high on the “Hostile Sexism Scale” (Glick and Fiske, 1996) rating them as relatively likely to violate sexual norms, including committing adultery (Stafford and Kline, 1996; but see Forbes et al., 2002) – presumably, an act that can lead to divorce. In a study of married, Catholic, American women, any non-traditional marital surnaming practice was seen by some as indicating the intention to leave the marriage, or self-centeredness (Suter, 2004). A study of college students in the US found that women who retain surname at marriage were seen as less committed to the marriage (Robnett et al., 2016). Based on these results, there are beliefs of ties between (1) women’s childbearing within marriage and activities or stances that may lead to divorce, with (2) marital surname change practice.

In the current study, wives who took their husbands’ surnames are compared with wives who did not, to assess whether the former had more children and longer marriages. These putative predictors and outcomes are all available in divorce files and perhaps not, together, elsewhere. These predictors moreover comprise easily accessed demographic data: Should they show themselves predictive of marital duration, and/or number of children of the marriage, then (after validation) they could be used to predict such data. This, in turn, could help communities and professional caregivers better plan services, depending on whether these communities will on average need more housing for larger families and/or for new divorcé(e)s and their families, or whatever.

That an apparent sacrifice (of name) is being made in the long absence of legal force suggests that, perhaps, something is gained by brides doing it. To the extent it may “… mark women as to ownership …,” as alluded to by Wilson and Daly (1992, at p. 315), it may be done by the bride to please a husband who views her as his, and/or to fetter her own future ability to portray herself as not her husband’s wife. To the extent the wife’s new surname is used publicly, it may constitute a repeated, public declaration of the union and may be viewed by witnesses as a sign of commitment to the husband. Further evidence this may be the case is comprised of the facts that (1) any re-partnering of the (ex-)wife might be impaired, as the fact she has an (ex-)husband would be apparent every time her name (plus honorific) was spoken, and (2) any remarriage of hers might necessitate yet further, public action – to again change surname – that would come at additional costs.

If name change indexes a woman’s marital commitment or is so construed, at least two hypotheses follow. That is, in addition to marital duration being predicted by the practice, women for whom the husband’s investment in children would be particularly advantageous (e.g., women planning more children), may view surname change especially favorably.

*Hypothesis* 1: The marriages of opposite-sex couples in which the woman took the man’s surname will be of greater duration.
*Hypothesis* 2: There will be a greater number of children produced within opposite-sex marriages in which the woman took the man’s surname.

## Materials and Methods

### Characteristics of Geographical Area

A sample of 2013–2014 divorce files on opposite-sex marriages from the Elgin County, Ontario, Canada, Superior Courthouse was searched. This courthouse houses all court files for all divorces within the county (chosen as the more accessible of the only two for which permission was granted for a search). Since the ability to search divorce files is limited, demographic characteristics of this county, along with the analogous data for Canada as a whole are provided in Table 1. This is so readers will be able to hypothesize whether results may apply in other jurisdictions given their demographic (dis)similarity. Unless otherwise noted, all data concerning Elgin County’s residents and analogous data for Canada as a whole pertain to 2011; the nearest census year to 2013–2014. This study was exempt from ethical review.

**Table 1:** Demographic data regarding Elgin County, Ontario, Canada, and Canada as a whole.

Parameter	Elgin County	Canada as a whole
Married or Live with a common-law partner	44,185^a^ (62%)	16,084,490^b^ (58%)
Married and Not Separated	38,035 (54%)	12,941,960 (46%)
Separated	2,230 (3%)	698,240 (2%)
Divorced	3,750 (5%)	1,686,035 (6%)
Immigrant to Canada	13%^c^	20%^d^
Unemployment	9%^e^	8%^f^
Average Yearly Individual Income, 2010 ($CDN)	35,265^g^	38,700^h^
Median Yearly Individual Income, 2010 ($CDN)	28,183^g^	29,859^i^
Average Yearly Household Income, 2010 ($CDN)	69,158^j^	84,900^k^
Median Yearly Household Income, 2010 ($CDN)	60,175^j^	66,000^k^
Percentage Households Spending >30% Combined Income on Shelter	24.2%^l^	25.2%^m^

All data are for 2011 unless otherwise noted.

a Of a total population 15 years of age or over of 70,755 (Statistics Canada, 2011a).

b Of a total population 15 years of age or over of 27,869,345 (Statistics Canada, 2017).

c Of 85,870 residents of Elgin County residents providing data (Statistics Canada, 2013a).

d Of those providing data (Statistics Canada, 2011c,d).

e Of 69,205 residents 15 years of age or over providing data (Statistics Canada, 2011a).

f Of residents 15 years of age or over providing data (Newfoundland and Labrador Statistics Agency, n.d.).

g Of 69,205 residents 15 years of age or over providing data (Statistics Canada, 2013a).

h Source: The Canadian Press, 2013.

i Source: Statistics Canada, 2011d.

j Of 33,485 private households providing data (Statistics Canada, 2013a).

k Source: Canada Mortgage and Housing Corporation, n.d.

l Of 32,375 private households providing data (Statistics Canada, 2011b).

m Source: Statistics Canada, 2013b.

Approximately 180 divorces are finalized in this county annually (personal communication: Melissa Kirby, Supervisor of Court Operations, Elgin County Courthouse, 8 September 2014). Elgin County spans 1,880.90 square kilometers (total population of 87,461: Statistics Canada, 2012), and is comprised of a mid-sized city, a town, and six smaller townships and municipalities.

### Data Characteristics and Search Technique

The searched divorce files tended to include the number and surnames of children, at least if these were minors. Each file included the first and surnames of the divorcing couple, their ages at marriage, and marriage duration (i.e., date of marriage to date of separation: full details available on request).

Each file also included the parties’ Marriage Certificate, always indicating the premarital surname of the bride and groom. Given the files contained only information from the date of marriage, the criterion for each divorcing wife being deemed to have undergone marital surname hyphenation or retention was any mention in the court divorce file of her using her premarital surname hyphenated with that of her husband or alone, prior to the date of separation. Note that every page of the divorce file, except where it is noted as having been searched merely cursorily, below, was searched for such premarital or hyphenated name. Divorcing women who assumed (the norm in Ontario, Canada: MacEacheron, 2016a) rather than legally changed surname at marriage, however, could have used their premarital surname within some pre-separation court file documents only. This may have been despite using a husband’s surname at all other times prior to separation (see generally Friess, 2007; Snyder, 2009, for extremely low occurrence of opposite-sex marriage grooms’ surname change).

The literature is nearly silent as to the proportion of women or men who take their spouse’s surnames in same-sex marriages (but see Underwood and Robnett, 2019). Due to the concern there would be insufficient numbers of same-sex divorces to afford adequate statistical power for analyses, and due to the very limited search time available, only opposite-sex divorces were examined.

A large effect size was expected, based on large effect sizes being observed in previous, quantitative research concerning marital surname change (MacEacheron, 2011, 2020), the finding in previous qualitative work that marital surname choice is sometimes a fraught decision (Boxer and Gritsenko, 2005), and the author judging the consequences of women’s marital surname choice to be high stakes. One such consequence is the surnaming of the children of the marriage solely for the father more often where the mother took the father’s surname at marriage (Johnson and Scheuble, 2002; Duchesne, 2006) – something about which husbands have been anecdotally reported to care deeply (Cherlin, 1978).

Note that in the quantitative literature on women’s marital surnaming, brides who kept their surname are usually grouped with those who hyphenated, and compared with those who took their husband’s surname (without keeping their own, too: see review in MacEacheron, 2016a). The decision was taken to do so here, too, for two reasons. First, doing so would make results more easily interpretable alongside such literature. Second, doing so would help ensure adequate number of wives in each group to be compared, so that statistical tests’ requirements would be met.

Two business days were granted to complete the current study’s file search. Thus, a method for sampling the divorce files was devised. One such procedure would have consisted of searching divorce files for a single year or other period only, or some proportion (1/n) of these, with each n^th^ file chosen for review. The proportion (or number) chosen for searching, however, would have had to allow for searching at least 26 × 2 divorce files to allow for 0.8 statistical power in *t*-testing (with two groups) at alpha = 0.05, with a large effect size, assuming each group contained at least 26 data points (Cohen, 1992). Assuming Johnson and Scheuble’s (1995) US finding that 4.6% of wives in the said country currently retain or hyphenate their surname at marriage applied to couples divorcing in Elgin County however, the wife would be expected to have retained her premarital surname in only one out of 26 divorce files. To obtain 26 files in which the wife retained or hyphenated her premarital surname, approximately 565 files would thus need to be searched. This approach was not feasible given the time allowed.

As such, the number of files feasible to search in 1 day (all then-finalized 2014 divorces the files of which were not being used by court staff: 108) was cursorily pre-searched simply to ascertain in how many the wife had either retained or hyphenated surname (see Table 2). Adequate statistical power at expected, large effect size given the other statistical parameters used was achieved. That is so, as 26 of the 108 divorce files were of couples the wife of which had retained or hyphenated surname. As such, only the just-noted 108 were searched in detail. As many additional files (*n* = 59) as could be searched in the remaining time allowed were searched cursorily, in reverse chronological order by divorce finalization date. Thus, all files not being used by court staff during the search, with divorce finalization dates between June 12, 2014 and October 15, 2013 were completely or cursorily searched: a total of 167 files. (Note that five files were in use by court staff during the search). Searches were confined as much as possible in time, as the passage of time might make marriages and/or divorcing wives and/or divorcing husbands less comparable among themselves. Files searched (on September 11 and 12, 2014) were the most recent ones available.

**Table 2:** Sample descriptive statistics: Out of total number of couples’ divorce files searched (*N* = 167), number providing data of each type.

Total	167	
**No. wives undergoing marital surname change**	**126** (75.45%)	
**No. wives retaining surname**	**38** (22.75%)	} 24.55% retain/hyphenate
**No. wives hyphenating surname**	**3** (1.80%)	
No. files searched in-depth	108	
No. husbands changing/hyphenating surname	0	
No. files with data on children (of marriage or not^*^)	107	
*No. citing 0 children*	*34 (31.78%)*	
*No. citing 1 child*	*26 (24.30%)*	
*No. citing 2 children*	*34 (31.78%)*	
*No. citing 3 children*	*10 (9.34%)*	
*No. citing 4 children*	*3 (2.80%)*	
*No. children not surnamed solely for husband*	*1 (0.74%)* ^∗∗^	
No. files with data on children of the marriage	108	
*No. citing 0 children*	*37 (34.26%)*	
*No. citing 1 child*	*24 (22.22%)*	
*No. citing 2 children*	*34 (31.48%)*	
*No. citing 3 children*	*10 (9.26%)*	
*No. citing 4 children*	*3 (2.78%)*	
No. files with wife’s income yearly data	42	
*Range ($CDN)*	*$0.00–$134,836.08*	
*Average ± SD ($CDN)*	*$24,932.47 ± $33,530.22*	
*Median ($CDN)*	*$11,057.86*	
No. files with husband’s yearly income data	45	
*Range ($CDN)*	*$0.00 – $305,273.12*	
*Average ± SD ($CDN)*	*$63,737.13 ± $50,214.44*	
*Median ($CDN)*	*$57,100.00*	
No. files with wife’s age^∗∗∗^	108	
*As of date of marriage:*		
*Range (years)*	*19–50*	
*Average ± SD (years)*	*30.63 ± 6.68*	
*Median (years)*	*28*	
*As of date of separation:*		
*Range (years)*	*24 – 59*	
*Average ± SD (years)*	*40.92 ± 8.85*	
*Median (years)*	*41*	
No. files with husband’s age	108	
*As of date of marriage:*		
*Range (years)*	*21 – 62*	
*Average ± SD (years)*	*33.33 ± 7.62*	
*Median (years)*	*32*	
*As of date of separation:*		
*Range (years)*	*24 – 62*	
*Average ± SD (years)*	*43.57 ± 9.14*	
*Median (years)*	*42*	
*Average greater age of husbands (years ± SD)*	*2.66 ± 7.62*	
*Median greater age of husbands (years)*	*2*	
No. files with marital duration (date of marriage to separation)	108	
*Range (days)*	*62 – 11,887*	
*Average ± SD (days)*	*3,887.18 ± 2,839.69*	
*Median (days)*	*3,121*	
*Range (years)*	*<1–32*	
*Average ± SD (years)*	*10.14 ± 7.79*	
*Median (years)*	*8*	
No. wives previously divorced	16 (9.60%)	
*No. previously changing surname*	*8 (4.80%)* ^∗∗∗∗^	
No. files searched cursorily	59	

∗ Administrative error led to no datum being recorded as to whether there were any children who were not children of the marriage, for one couple. This couple had no children of the marriage (though each, separately, may have had other children – such as children born prior to the marriage). Only two children out of the total of 136 (1.47%) mentioned in the divorce files were not children of the marriage being dissolved. One bore as his or her sole surname that of the relevant divorcing husband: No datum was ascertainable from the relevant file as to the surname of the other.

∗∗ Only one child out of the total of 136 (0.74%) did not bear solely the surname of the divorcing father. The divorcing woman (presumably, its mother) listing this child had retained her premarital surname. This child bore a hyphenated surname, combining his or her (presumed) mother’s with that of the man she was divorcing from (presumably, the child’s father).

∗∗∗ Complete data were available as to couples’ ages, for the portion of divorce files searched in-depth (*n* = 108). The separation date for one such marriage was not recorded due to administrative error. Thus, because marriage duration (i.e., time between separation date and wedding date) was used to assess age as of date of marriage, not only the duration of one marriage, but also spouses’ age as of data of marriage, is missing for one divorcing couple (file).

∗∗∗∗ Of the 16 divorce files the wives in which had previously divorced, data regarding *previous* marital change/retention/hyphenation was missing in three. Out of the 13 cases remaining, eight wives had changed surname previous to the marriage which was the subject of the divorce file (see above).

Where children’s birthdates were specified, only if such birthdates were on or following the date of marriage or if these children were specified as the children of both parties in other file documentation, were such children considered children of the marriage. Where children’s birthdates were not specified, it was assumed they were children of the marriage, due to these children’s existence being relevant enough to the divorce proceedings to list them.

### Statistical Methods

Planned analyses included *t*-test and two regressions, as follows. *t*-tests assessed whether marriages in which the wife took the husband’s name were associated with younger bridal age and/or longer duration. A regression analysis with number of children as dependent variable (DV) was conducted with the (effect-coded) predictor of whether the wife changed surname versus not (hyphenation included in the latter category), and control predictor of marriage length, to further test the hypothesis that marital surname change predicted number of children of these marriages. Marriage length was included as a covariate because it logically may, independently of whether or not the wife underwent marital surname change, predict number of children of the marriage, given that human conception and gestation require non-negligible amounts of time. In a second regression, length of marriage comprised the DV, the (effect coded) predictor was the ex-wife having undergone marital surname change or not, and bride’s age served as co-variate (Given that wife’s [or husband’s] age at time of marriage might reasonably be related to ultimate marriage duration, and given that wife’s age has been previously associated with her decision to change versus retain/hyphenate her surname at marriage, it was included as covariate.)

Divorce file data have a degree of reliability based on the fact they are publicly presented to the court which has the power to demand official records, by parties who are equal before the court (estranged husband and wife) with sometimes opposing interests as to what those data should be. These opposing interests stem from, for example, possessing either an interest in paying less or in receiving more child support funds. Each party has the right if not the obligation to present data on, for example, incomes, with the (disinterested) court reviewing them and coming up with income figures based in part or in whole on them (Government of Canada: Department of Justice, 2018).

## Results

### Descriptive Statistics

A total of 167 divorce files were searched: *n* = 108 in-depth, and *n* = 59 cursorily.

In 33 divorces, the wives had either hyphenated or retained premarital surname, and in the remaining 75, the wives had changed surname to that of their husbands. The proportion of children not receiving solely their father’s surname did not differ depending on whether their mothers had taken their husbands’ surnames (*t* [17] = 1.00, *p* = ns). (Note that this *t*-test must be considered underpowered: Cohen, 1992).

### Association Between Surname Retention Versus Change, and Number of Children

Of the wives who had either retained their premarital surnames or hyphenated these with those of their husbands, average number of children of the marriage was 0.94 (±1.10). Of the remaining wives, average number of children of the marriage was 1.37 (±1.10).

Now reported is the planned regression with number of children of the marriage as DV, (effect-coded) predictor of wife undergoing surname change versus not (hyphenation included in the latter category), and covariate of marriage length, to test the hypothesis that marital surname change predicted number of children of these marriages. In an attempt to attain a good model fit, two different regressions appropriate to a DV of count data – Poisson and Negative Binomial -‐ were conducted. The two regressions’ Akaike information criterion (AIC) and Bayesian information criterion (BIC) values were compared, to determine which regression achieved greater fit to the data. Lower AIC and BIC values in the Poisson (AIC = 293.86, BIC = 326.71) than in the Negative Binomial modeling (AIC = 301.88, BIC = 334.73) showed better fit of the former. Thus, the former is reported (see Table 3).

**Table 3:** Poisson regression, DV = number of children of the marriage (*n* = 107).

Parameter	b	SE b	Wald	df	*p*	Exp(b)
Intercept	−0.40	0.20	3.84	1	0.05	0.67
Wife’s surname choice – *changed name*	0.10	0.21	0.22	1	0.641	1.10
Wife’s surname choice – *retained/hyphenated name*	0^a^					1
Marriage duration (years)	0.05	0.01	19.56	1	0.000	1.05

a Set to 0 because it is a redundant parameter. In 30% of marriages (effect coded 1), the wife had either retained her pre-marital surname or hyphenated it with that of her husband.

The relevant value of exponentiated b (incidence rate ratio) shows that every year of greater marriage duration is associated with a predicted 5% increase in number of children of the marriage. Wife’s surname choice was not significantly predictive.

### Association Between Surname Retention Versus Change, and Duration of Marriage

Duration of marriage was not determinable for one divorce file in which it was searched, due to administrative error. Of the remaining (*n* = 32) marriages the wives in which had either hyphenated their premarital surname with that of their husbands or retained their premarital surname, average marriage duration was 2,639.00 days (±2,353.04), or 6.78 years (±6.45). Of the marriages the wives in which had changed their surnames to those of their husbands (*n* = 75), average marriage duration was 4,419.73 days (±2,875.82), or 11.57 years (±7.90). A *t*-test comparing duration of marriage between the former and latter demonstrated greater such duration in the former: *t* (105) = −3.09, *p* = 0.002, Cohen’s *d* = 0.63 or large. Note that as the relevant prediction was directional (i.e., that the former group’s marriage duration would be less than that of the latter’s), one-tailed testing was used.

Now discussed are the results of Poisson and Negative Binomial regressions with marriage duration in years as DV, (effect coded) predictor of wife undergoing surname change versus not (hyphenation included in the latter category), and covariate of wife’s age at time of marriage. The two regressions’ AIC and BIC values were compared, to determine which modeling achieved greater fit. Lower such values in the Negative Binomial (AIC = 717.13, BIC = 725.15) than in the Poisson modeling (AIC = 968.61, BIC = 976.63) showed better fit of the former. Thus, the former will be reported here (see Table 4).

**Table 4:** Negative binomial regression, DV = duration of marriage in years (*n* = 107).

Parameter	b	SE b	Wald	df	*p*	Exp(b)
Intercept	2.83	0.53	28.85	1	0.000	17.01
Wife’s surname choice – *changed name*	0.47	0.23	4.31	1	0.038	1.60
Wife’s surname choice – *retained/hyphenated name*	0^a^					1
Wife’s age at time of marriage	−0.03	0.02	3.62	1	0.057	0.97

a Set to 0 because it is a redundant parameter. In 30% of marriages (effect coded 1), the wife had either retained her pre-marital surname or hyphenated it with that of her husband.

Consistent with the relevant *t*-test result, the wife’s surname choice was significantly predictive of marriage duration (Wald’s *χ*^2^ = 4.31, *df* = 1, *p* = 0.038), with having changed surname to that of the husband predicting greater such duration. As can be seen from the value of exponentiated b associated with wife’s surname choice, women who changed surname had marriages of approximately 60% greater duration than women who retained their premarital surnames or hyphenated. Wife’s age at time of marriage, on the other hand, was a marginally significant, negative predictor of marriage duration (Wald’s *χ*^2^ = 3.62, *df* = 1, *p* = 0.057).

## Discussion

A strong finding was that marriages in which women took their husbands’ surnames lasted longer than marriages in which women did not. All else equal, given the greater number of years a married couple are together the more children are possible and reasonably expectable, number of years together should be an important predictor of number of children of marriages. Thus, to an extent, where women’s marital surname choice predicts marital duration, such choice might, should this result prove generalizable, be used to help predict number of children of marriages.

Marital surname change did not predict, however, the number of children of the marriage, within a regression using that and marital duration as predictors. This could simply be due to the fact that marital duration is more predictive. Note that a planned *t*-test comparing the number of children of the married couples where the wife had kept or hyphenated her surname at marriage, versus those who had changed their surname to that of their husband, arguably showed a greater number of children for the latter: *t* (106) = −1.90, *p* = 0.03, Cohen’s *d* = 0.39 or moderate (As my prediction was directional [i.e., that the former group of women would have fewer children of the marriage than would the latter], one-tailed testing was used). Note that this is merely an “arguable” finding, as without a *large* effect size, this analysis must be considered underpowered (Cohen, 1992). Women’s marital surname choice, after any successful validation of its predictiveness of marital duration, might be used to predict communities’ needs for divorce-relevant services (e.g., courtroom space, counseling), at the times these would tend to be needed. To the extent marital duration, again after any successful validation of its predictiveness, predicts number of children of marriages, the needs related to the number of children in a community (e.g., size of typical housing needed) may be better anticipated. As noted, the predictor of women’s marital surname choice could comprise relatively easily accessed demographic data. This means it might easily be used as a predictor, as above.

It is speculated that a major piece of the puzzle of women’s continued marital surname change, long past legal necessity and cultural novelty of retention/hyphenation, is that marriage may be understood as fundamentally a reproductive union (Buckle et al., 1966). It is this context in which children are raised, notwithstanding the tremendous historical and cross-cultural variability in the expectations and practices associated with marriage (Murdock, 1949). That women’s marital surname change is done at the start of the bride and groom’s reproductive union, may indicate that it pertains to that reproductive union. Previous social scientists studying women’s marital surname choice have never done so under the lens of regarding marriage as a reproductive partnership. When marriage is viewed this way various, special hypotheses as to ultimate and proximal reasons for women’s choice of marital surname arise. Women’s marital surname change, as a public declaration of the union, may be construed as a signal of commitment by the bride to the groom. If this is true, it would follow that brides who particularly require investment by their future husband, for example because they intend on having more children, would regard women’s marital surname change more positively and engage in it more.

Whether children born to opposite-sex parents bear their father’s, mother’s, or a combination surname affects the continuation of such names, and thus may be considered important. Here, the receipt by any child of a surname other than solely that of the presumed father, was a practice of negligible frequency. Women’s marital surname change, given it is more frequently followed by children of the marriage being surnamed for the woman’s husband (Johnson and Scheuble, 2002; Duchesne, 2006), would additionally signal intention to surname children of the marriage for the husband, and might constitute an attempt on the part of a bride to enhance paternal investment (see MacEacheron, 2016a). Desire for investment on the part of the wives, particularly, might be explicable by greater nutritional and other resourcing needs associated with pregnancy and lactation (e.g., Abdulla and Abdulla, 2004) and women’s average income drops associated with childcare (see Cain Miller, 2014: note this may especially be true for women desiring a higher number of children), as well as by paternity uncertainty. Women might also particularly benefit from resource-investment because mothers, in multiple cultures, are the relatives providing the most care for children (e.g., in industrial societies, Minge-Klevana, 1980). (Again, this may ultimately be due to paternity uncertainty, and thus a child’s mother being its only certain, genetic parent.) Thus, mothers may tend to have less time than any other class of children’s relatives, to procure resources for themselves and for their children, and yet may be responsible for direct provision of such resources to the latter.

The current male, romantic partner of a mother, as closest (along with her) putative, genetic relative of such child, is a strong candidate for investor in that child. That is so, since such investment augments the father’s reproductive success – albeit only if he is the genetic father. Women entering reproductive unions with men therefore, where it is particularly in those women’s interest to elicit such support (e.g., if desiring more children), may attempt to provide more paternity assurance than other women. One way, again speculatively, would be to demonstrate commitment to him/the marriage, by undergoing the costly signal of marital surname change.

### Limitations

The within method of determining whether a divorcing wife had either retained or hyphenated premarital surname likely entailed overestimation of the frequencies of these practices. No better estimation method seems devisable, however, given the absence of any other North American records of women’s marital surname retention/hyphenation/change, which include all of bridal age, marriage duration, number of children, and children’s surnames (see generally Cherlin, 1978; Goldin and Shim, 2004). Regardless, because some women who had changed surname at marriage were almost certainly erroneously included as retainers/hyphenators, predictions herein would tend to be *less* supported. Public records that cover all instances of a phenomenon (e.g., all divorces in a county), additionally, tend to possess ecological validity to the extent they are reliable – as divorce records may especially be, given the adversarial nature of presentation of data within, sometimes verified by disinterested courts (see generally Government of Canada: Department of Justice, 2018).

Divorcing spouses would seem to not be representative of spouses in general. It is reasonable to question whether data from those divorcing should be used to assess commitment (which, if unequivocal, would result in never divorcing). In other words, it may be that only spouses (including wives) with lesser marital commitment may be compared with one another *via* this study. Given, however, that a divorce is granted including where only one spouse wants it, and this spouse is at least sometimes the husband, divorcing wives could not solely represent wives with lesser marital commitment. 42.1% of marriages celebrated in 2008 in Ontario are projected to end in divorce within 30 years (Statistics Canada and Kelly, 2012). Given many marriages even of relatively long duration end in divorce, it is submitted that couples studied were not only representative of couples, or husbands or wives, of lesser marital commitment, at least at the inception of the marriage (when name change presumably mostly occurs). One implication of the presumed existence of at least some husband-initiated and/or mostly husband-driven divorce on the current data, when considering the finding that marriages the wife in which did not take the husband’s surname did not last as long, is that husbands may be tending to drive or partially drive divorce, preferentially where their wives did not take their surnames.

Note the partial, basic explanation of women’s marital surname change as a costly signal of commitment described in this article, is fully compatible with another explanation a reviewer suggested, which might predict the same outcomes found. That suggestion, was that people possessing traditional beliefs concerning gender and marriage, would tend to assortatively mate (and marry one another). This article seeks, however, to provide a more ultimate explanation than that assortative mating (on relevant beliefs) exists. Indeed, no hypothesis *why* assortative mating on traditional beliefs may exist has been, to the author’s best knowledge, quantitatively tested in the literature on women’s marital surname change, nor has the existence of such assortative mating even been empirically documented. It is even arguable that the fact that those with traditional beliefs concerning women’s marital surname change and delaying or avoiding divorce may seek each other for marriage is not a potential *explanation* for women’s marital surname change being associated with staying in marriages longer, but is instead a (potentially very valuable) *observation* that (1) these two beliefs tend to co-occur in individuals, and that (2) those with such beliefs tend to marry one another.

### Possible Future Directions

The 24.55% share of divorcing wives in the within sample who did not change their surnames solely to that of their husbands appeared high compared with figures of 6 and 4.6% of Johnson and Scheuble (1995) and Gooding and Kreider (2010), respectively, representative of US, married, *non-divorcing* women. On the other hand, the figure 24.55% appears similar to the analogous percentage of Canadian destination brides to Hawai’i in 2006 either keeping or hyphenating surname (25.22%: MacEacheron, 2011). Assuming Canadian (or North American) representativeness of the current study’s data, it is posited that (a) differences over time, and/or (b) cultural differences between Canada and the US (which might be assumed based on historical, geographical, and sometimes linguistic differences), might be partly explanatory. It is also possible that among the divorcing couples studied, marriages in which wives retained or hyphenated surname were oversampled – disproportionately more marriages where women did not take their husbands’ surnames ended in divorce in the given time period and area studied, compared with other such marriages. Current replication of the Johnson and Scheuble (1995) study in the US and Canada, would uncover whether (a) is operating. The possibility of (b) could be partially assessed *via* surveying the attitudes towards women’s marital surname change, retention, and hyphenation, in both countries. To assess whether women’s marital surname change remains as a strong predictor of marital duration among those going on to divorce where underlying culture differs from that of Canada, it may be useful to repeat the current study in countries in which women may change surname at marriage, but less commonly do.

It would seem possible to test whether marriages in Elgin County in which women did not take solely their husbands’ surname were more likely to end in divorce than were other marriages within the time period studied. This could be accomplished by surveying women within Elgin County who were spouses in intact marriages within that period, as to whether they took (solely) their husbands’ surname at marriage. Then, this rate of surname change versus retention/hyphenation would be compared with that of only the divorcing wives in the current study. Note, however, that there is no publicly available registry of women within intact marriages in Elgin County (or anywhere in Canada), nor even one just of women, to whom such a survey could be sent. Although there are directories of marriage certificates issued in Canada (e.g., in Ontario, administered by ServiceOntario), it is only possible to request a search for a marriage certificate of two *given* individuals married to each other (i.e., whose full names are provided: e.g., ServiceOntario, 2012–2016). Additionally, there seems to be no likely source of Elgin County’s women’s names, as such publicly available name listings as telephone directories may contain only a male householder’s name or an initial.

## Conclusion

After adequate replication, marital surname change versus retention/hyphenation might be used by demographers as one predictor of marital duration among those going on to divorce, and/or as a tool to help predict the number of children expected to be produced from such marriages. Such predictions could assist those planning the level of services to provide to (divorcing) spouses and their children. At least within the studied county and time period, the finding of greater marriage duration among opposite-sex (divorced) couples the wives in which took the husbands’ names, is consistent with (1) marital surname change comprising one, possible signal of brides, regarding intention to remain in their marriages longer/under a greater set of circumstances, and/or divorce-relevant actions within the marriage, and/or (2) wives’ marital surname choice impacting (i) which husbands married them, such that *husbands* with greater intention to remain in their marriages longer/under a greater set of circumstances married women indicating they would take the husbands’ surnames, and/or (ii) husbands’ divorce-relevant actions within the marriage.

## Data Availability Statement

The dataset presented in this article is not readily available, because divorcing parties’ names must be presented in person at the divorce file repository, before these are provided for inspection. Requests to access the dataset should be directed to Maretta Miranda, Counsel, Ministry of the Attorney General (Ontario), Toronto, Canada.

## Ethics Statement

Ethical review and approval was not required for the study on human participants in accordance with the local legislation and institutional requirements. Written informed consent for participation was not required for this study in accordance with national legislation and institutional requirements.

## Author Contributions

The author confirms being the sole contributor of this work and has approved it for publication.

### Conflict of Interest

The author declares that the research was conducted in the absence of any commercial or financial relationships that could be construed as a potential conflict of interest.
